# The genie in the bottle-magnified calcium signaling in dorsolateral prefrontal cortex

**DOI:** 10.1038/s41380-020-00973-3

**Published:** 2020-12-15

**Authors:** Amy F. T. Arnsten, Dibyadeep Datta, Min Wang

**Affiliations:** Department of Neuroscience, Yale University School of Medicine, New Haven, CT 06510 USA

**Keywords:** Neuroscience, Physiology

## Abstract

Neurons in the association cortices are particularly vulnerable in cognitive disorders such as schizophrenia and Alzheimer’s disease, while those in primary visual cortex remain relatively resilient. This review proposes that the special molecular mechanisms needed for higher cognitive operations confer vulnerability to dysfunction, atrophy, and neurodegeneration when regulation is lost due to genetic and/or environmental insults. Accumulating data suggest that higher cortical circuits rely on magnified levels of calcium (from NMDAR, calcium channels, and/or internal release from the smooth endoplasmic reticulum) near the postsynaptic density to promote the persistent firing needed to maintain, manipulate, and store information without “bottom-up” sensory stimulation. For example, dendritic spines in the primate dorsolateral prefrontal cortex (dlPFC) express the molecular machinery for feedforward, cAMP–PKA–calcium signaling. PKA can drive internal calcium release and promote calcium flow through NMDAR and calcium channels, while in turn, calcium activates adenylyl cyclases to produce more cAMP–PKA signaling. Excessive levels of cAMP–calcium signaling can have a number of detrimental effects: for example, opening nearby K^+^ channels to weaken synaptic efficacy and reduce neuronal firing, and over a longer timeframe, driving calcium overload of mitochondria to induce inflammation and dendritic atrophy. Thus, calcium–cAMP signaling must be tightly regulated, e.g., by agents that catabolize cAMP or inhibit its production (PDE4, mGluR3), and by proteins that bind calcium in the cytosol (calbindin). Many genetic or inflammatory insults early in life weaken the regulation of calcium–cAMP signaling and are associated with increased risk of schizophrenia (e.g., *GRM3*). Age-related loss of regulatory proteins which result in elevated calcium–cAMP signaling over a long lifespan can additionally drive tau phosphorylation, amyloid pathology, and neurodegeneration, especially when protective calcium binding proteins are lost from the cytosol. Thus, the “genie” we need for our remarkable cognitive abilities may make us vulnerable to cognitive disorders when we lose essential regulation.

## Introduction

The primate cortex performs an extraordinary number and range of operations decoding sensory events, integrating sensory inputs with previous experience, and representing information in higher networks independent of sensory stimulation to create our “mental sketch pad” and generate goals for action. We are learning that the cortical networks that subserve these varied functions also have differing circuit architectures and unique molecular needs. For example, the newly evolved dorsolateral prefrontal cortex (dlPFC) contains microcircuits in deep layer III with extensive recurrent excitation that generate the persistent neuronal firing needed to maintain mental representations in working memory. Emerging data suggest that the molecular mechanisms needed to sustain persistent firing in dlPFC are very different from those required to accurately decode a sensory stimulus in primary visual cortex (V1). Most importantly, the molecular signaling events needed to sustain mental representations also appear to render these circuits especially vulnerable to dysfunction when errors emerge from genetic and/or environmental insults. In particular, we propose that the magnification of calcium signaling needed for the persistent firing of dlPFC microcircuits confers vulnerability to atrophy and degeneration when regulation is lost, e.g., by inflammation. The current review describes the differences in neurotransmission and neuromodulation between newly evolved vs. primary sensory cortices, and then speculates on how these disparities may underlie the susceptibility of dlPFC circuits in mental disorders.

## Hierarchies in timescales and vulnerabilities across primate cortex

**Fig. 1:**
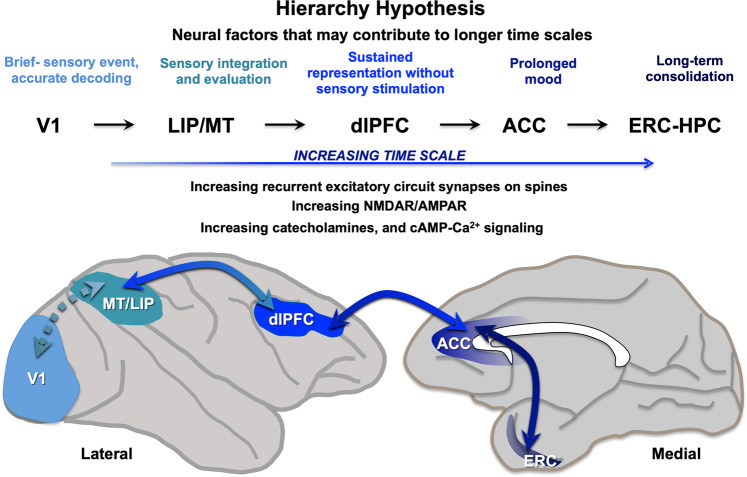
Increasing timescales and information processing across the cortical hierarchy. Computational analyses of monkey and human data indicate a hierarchy of timescales across the cortex, consistent with the increasing integration, representation, and consolidation of information as one ascends the hierarchy. For example, analyses of the intrinsic fluctuations in neuronal spiking from rhesus monkeys performing cognitive tasks show that sensory cortical areas have shorter timescales (~65–75 ms), PFC association areas have longer timescales (~125–200 ms), the ACC still longer (~250–350 ms), and the entorhinal cortex longest of all, with a range of timescales up to 20 s, consistent with its collaborative role with hippocampus in longer term memory consolidation [4, 11]. As reviewed here, there are also gradients in the degree of recurrent circuits with excitatory synapses on spines, as well as an increasing ratio of NMDAR/AMPAR transmission, catecholamine innervation, and calcium–cAMP actions that permit higher cognitive operations, but also confer vulnerability to dysfunction with environmental and/or genetic insults. Cortical areas are shown on a lateral and medial view of a rhesus monkey brain: V1 primary visual cortex, MT middle temporal cortex (visual area V5), LIP lateral intraparietal cortex, dlPFC dorsolateral prefrontal cortex, ACC anterior cingulate cortex, ERC entorhinal cortex.

The hierarchical organization of the primate cortex has been appreciated for decades by neuroanatomists [1–3]. More recently, computational analyses have demonstrated a hierarchy in timescales across the cortex that is consistent with the emergence of cognitive and affective functions in higher areas (Fig. 1). Increasing timescales across the cortical hierarchy have been seen in the intrinsic fluctuations in neuronal spiking across cortical areas in nonhuman primate cortex [4], and in human MRI [5] and transcriptomic [6] data, whereby sensory cortical areas have shorter timescales, and PFC association areas have longer timescales. This idea is consonant with the functions of these cortical areas, where primary visual cortex (V1) would require a short timescale to accurately decode the onset and offset of a visual stimulus, sensory association cortices such as MT (middle temporal area V5) or LIP (lateral intraparietal area) would need longer timescales to integrate and analyze sensory information and compare them with stored patterns to facilitate recognition, and the dlPFC would need still longer timescales to maintain and manipulate information for many seconds without sensory stimulation [7]. The analyses of rhesus monkey data revealed still longer timescales from neurons in the anterior cingulate cortex (ACC), which integrate affective and cognitive information [8], and in humans, can contribute to sustained mood states [9, 10]. Finally, a recent analysis of monkey entorhinal cortex (ERC) revealed a range of timescales, including very long scales consistent with the ERC–hippocampal role in longer term memory consolidation [11].

A hierarchy in cortical processing is particularly relevant to our understanding of mental disorders, as there is a general correlation between circuits with longer timescales, and vulnerability in cognitive disorders. For example, there is a near perfect correspondence between the cortical hierarchy and the pattern and sequence of tau pathology in Alzheimer’s disease (AD), something noted by primate neuroanatomists and neuropathologists for decades [12–15]. Similarly, in schizophrenia, there is much greater dendritic spine loss in the dlPFC than in V1 [16], while the rostral cingulate areas are a focus of pathology in mood disorders such as depression [17–19]. Thus, understanding the differences in circuit architecture, neurotransmission, and neuromodulators along the cortical hierarchy may also help us understand the mechanisms that confer vulnerability to mental disorders.

There are important differences in neuronal morphology, circuit architecture, and physiological properties across the cortical hierarchy [20, 21]. These include factors that promote persistent neuronal firing needed for higher cognition, such as increasing local recurrent circuits with corresponding spine density [22–24] and increasing numbers of disinhibitory interneurons [25] in higher circuits. In primate dlPFC, these recurrent microcircuits are concentrated in deep layer III [26, 27], the focus of spine loss in schizophrenia [16], and a target of tau pathology in AD [28, 29]. The following review explores how increases in calcium signaling across the hierarchy promote persistent firing, with particular comparisons between area V1 with dlPFC, where there are striking differences consonant with their varied functions and susceptibility to atrophy.

## Differences in neurotransmission—AMPAR vs. NMDAR transmission

**Fig. 2:**
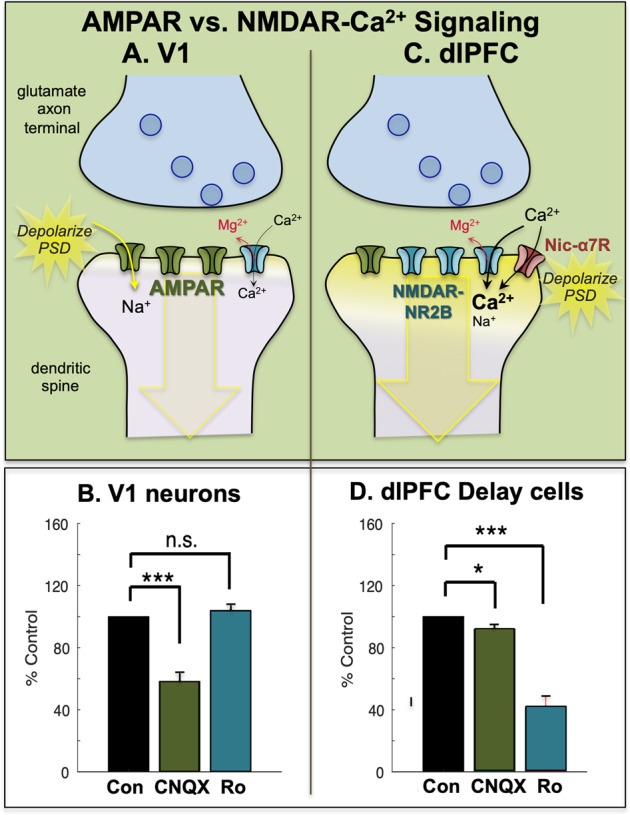
Differences in neurotransmission in primate V1 vs. dlPFC. **a** Neurons in primate V1 exhibit classic neurotransmission, relying heavily on glutamate stimulation of AMPAR with only a minor contribution of NMDAR. The rapid kinetics of AMPARs are appropriate for the accurate encoding of a sensory event. **b** The stimulus-evoked firing of V1 neurons in monkeys depends on AMPAR, as even low doses of an AMPAR antagonist (CNQX) greatly reduce firing. In contrast, equivalent doses of an NMDAR–NR2B antagonist Ro25-6981 (Ro) have little effect on V1 neuronal firing. **c** In contrast to V1, layer III dlPFC delay cells rely on NMDAR (NR2A and NR2B) with only subtle reliance on AMPA. The permissive depolarization of the PSD is instead contributed by acetylcholine via Nic-α7R (shown) and muscarinic M1R (not shown), both of which reside within the glutamatergic PSD [33, 34]. It is of interest that the Nic-α7R fluxes the most calcium of all cholinergic receptors, and that M1R can increase internal calcium release via Gq signaling. These data suggest that calcium entry from NMDAR–NR2B, as well as from Nic-α7R and M1R signaling, may maintain sufficient calcium near the PSD to maintain depolarization and allow persistent firing. M1R also enhance neuronal firing by closing KCNQ “m” channels, some of which are localized in the glutamate PSD [34]. These powerful cholinergic actions in dlPFC contrast with more subtle effects in V1, where nicotinic receptors are restricted to presynaptic thalamic inputs in layer IV, and to interneurons [180], and acetylcholine has a more classic, modulatory role, e.g., enhancing responses during attention [181, 182]. **d** Delay-related firing of dlPFC neurons in monkey depend on NMDAR, as even low doses of an NMDAR–NR2B antagonist such as Ro greatly reduce firing. In contrast, equivalent doses of an AMPAR antagonist CNQX have little effect on delay cell firing. **b** and **d** were adapted with permission from Yang et al. [30].

A comparison of the glutamate receptors mediating neurotransmission in primate V1 vs. dlPFC shows striking differences [30], where V1 neurons rely on AMPAR, while dlPFC circuits rely on NMDAR with NR2B subunits that flux high levels of calcium (Fig. 2). These data arise from physiological recordings from behaving monkeys, where small amounts of drug are iontophoresed (“electrically sprinkled”) onto the neurons being recorded. Monkey physiological data are consonant with transcriptomic data from human brain, indicating that neurotransmission varies widely across the cortex, and that there is an increasing reliance on NMDAR–NR2B as one ascends the cortical hierarchy.

### Rapid AMPAR neurotransmission in primate V1

Neurons in monkey area V1 that respond to visual stimuli have a great reliance on AMPAR transmission, where even low doses of AMPAR blockers such as CNQX markedly reduce stimulus-related firing [30] (Fig. 2a, b). In contrast, it requires very high doses of NMDAR blockers to reduce V1 neuronal firing [30] (Fig. 2b). A reliance on AMPAR stimulation is consistent with the function of V1 neurons, as the rapid kinetics of these receptors, in addition to their membrane properties [20], would allow accurate decoding of an event in the environment.

### Reliance on NMDAR–NR2B and acetylcholine in primate dlPFC

Neurons in the dlPFC have a very different function than those in V1: they must generate and sustain mental representations without sensory stimulation. Neurons with this capability to maintain persistent firing across the delay period in a working memory task are termed “delay cells.” Persistent firing of delay cells is thought to arise from extensive recurrent excitatory circuits mediated by NMDAR [31] (Fig. 2c, d), a finding predicted by computational models [32]. Thus, even low-dose blockade of NMDAR, including antagonists that selectively block those with NMDAR–NR2A or NMDAR–NR2B subunits, markedly reduces delay cell firing [32]. In classic circuits, NMDAR–NR2B are often found outside the synapse, but in layer III dlPFC, they are exclusively within the postsynaptic density (PSD), consistent with their direct mediation of neurotransmission [32]. Importantly, NR2B subunits close slowly and flux large amounts of calcium, which may be a key aspect of why they support persistent firing in computational models [32] and neurons [31].

In contrast to NMDAR, blockade of AMPAR has remarkably subtle effects on delay cell firing [31] (Fig. 2d). This finding was initially confusing, as it is generally thought that AMPAR are essential to depolarize the PSD membrane and relieve the magnesium (Mg^2+^) block within the NMDAR pore, permitting NMDAR actions (Fig. 2a). However, in dlPFC, this key permissive role appears to be played by acetylcholine acting at Nic-α7R and muscarinic M1R within the glutamate synapse [33, 34], which may depolarize the PSD to support persistent firing (Fig. 2c). These data are consistent with behavioral data showing that Ach depletion from dlPFC is as deleterious as removing the cortex itself [35]. As acetylcholine is released during wakefulness but not deep sleep, these mechanisms also help to coordinate cognitive state with arousal state, permitting conscious experience during wakefulness, but may render us unconscious during deep sleep when there is no acetylcholine release.

### NMDA-NR2B transmission increases across the cortical hierarchy

An increasing role for NMDAR–NR2B in dlPFC vs. V1 is likely true in human cortex as well, based on transcriptomic analyses. The NMDAR–NR2B is encoded by the *GRIN2B* gene, and GRIN2B expression increases across the cortical hierarchy from V1 to dlPFC to ACC in humans [6]. These data suggest that neurons in the ACC would be particularly dependent on NMDAR; however, there have been no iontophoretic recordings from monkey ACC to test this hypothesis. Data from rats show that NMDAR–NR2B in ACC mediate the sensitized response to pain [36], suggesting that these receptors may contribute to the long timescale seen in primate ACC neurons where affective information is integrated with cognitive information from dlPFC.

Extensive rodent data have demonstrated important roles for NMDAR in hippocampal function, where NMDAR contribute to grid cell firing in ERC [37], and have an essential role in mediating long-term potentiation (LTP) in CA1 and CA3 [38], including a role for NMDAR–NR2B in “metaplasticity” [39]. Calcium entry through NMDAR–NR2B is particularly important for initiating LTP, e.g., via CaMKII signaling [40]. ImmunoEM suggests a similar relationship in monkeys [41]. These data would be consistent with an increasing reliance on NMDAR, and particularly those with NR2B subunits and high calcium influx, at higher levels of the cortical hierarchy.

## Differences in neuromodulation across the hierarchy

There are also gradients in neuromodulators and signaling mechanisms across the cortical hierarchy. There are gradients in modulatory receptors [6], catecholamine innervation [42–45], and the expression of cAMP-related [46] and calcium-related proteins such as calbindin in pyramidal cells [47], which have low levels in primary sensory cortex but increase across the cortical hierarchy. For example, in vitro studies of rodent cortex find that calcium and cAMP can drive persistent neuronal firing in higher cortical areas such as the mPFC, ACC, and ERC [48–50]. Most of the research in primate has focused on comparisons between V1 vs. dlPFC, where these signaling pathways have differing subcellular localizations and physiological actions. For example, local application DA has no effect on V1 neuronal firing [51], but has a powerful inverted-U dose effect on dlPFC neurons [52, 53] through postsynaptic actions in spines, where cAMP–PKA signaling magnifies internal calcium release and opens potassium (K^+^) channels to reduce firing, the genie in the bottle [54]. These powerful, feedforward actions require tight regulation or toxic consequences ensue. The following is a brief review of the differences in modulatory influences across the cortical hierarchy, focusing on the two areas where these actions have been best studied, V1 and dlPFC.

### Classic cAMP actions in hippocampus and V1

The traditional neuromodulatory role of cAMP signaling is to strengthen synaptic connectivity, through both presynaptic and postsynaptic actions. For example, in rodent hippocampus, elevated calcium–cAMP–PKA signaling in spines can ultimately lead to phosphorylation of CREB, and transcriptomic events that strengthen existing synapses or even make new ones [55, 56]. As described above, this can be initiated through sufficient calcium entry through NMDAR [40], but also through internal calcium release from the smooth endoplasmic reticulum (SER) [57], which can internalize K^+^ channels and spur cAMP–PKA signaling [58], and elevate calcium entry into the nucleus to alter transcription [56]. Thus, calcium plays a role through many phases of plasticity [59]. LTP in dentate gyrus and CA3 neurons is also facilitated by NE stimulation of β-AR via increased cAMP–PKA signaling [60–62] that may occur to salient events, consolidating them in episodic memory. It is challenging to know if these same cAMP actions occur in primate hippocampus, but it is likely that these mechanisms are conserved.

**Fig. 3:**
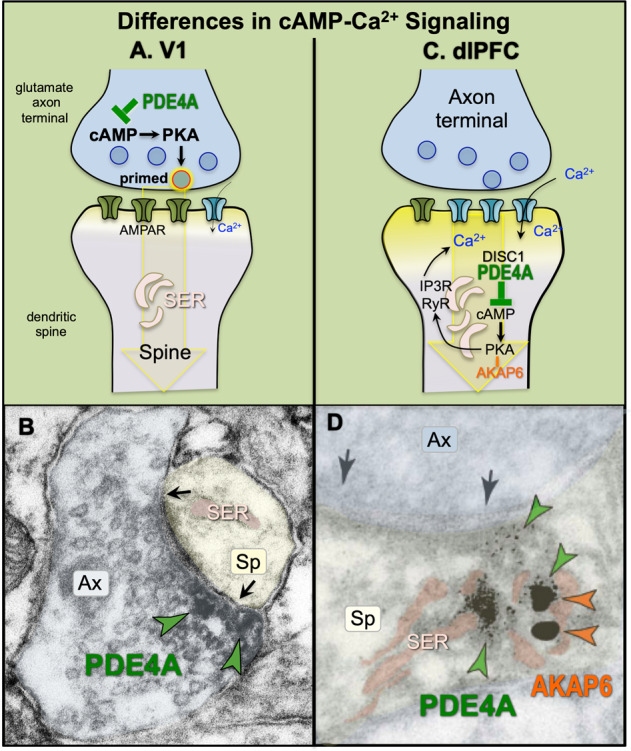
Differences in cAMP signaling in primate V1 vs. dlPFC. **a** Schematic diagram showing that cAMP signaling proteins are concentrated presynaptically in layer III of primate V1, where cAMP–PKA signaling primes vesicles for release. **b** Labeling for the phosphodiesterase, PDE4A, which catabolizes cAMP, is concentrated in glutamate-like axon terminals in V1 layer III, often surrounding synaptic vesicles. Labeling is indicated with green arrowheads. Parallel physiological studies found that activation of cAMP signaling in V1 increased stimulus-evoked neuronal firing, consistent with cAMP–PKA signaling enhancing glutamate release [30]. Image adapted from Yang et al. [30] with permission. **c** Schematic diagram showing that cAMP signaling proteins are concentrated postsynaptically in layer III of primate dlPFC, positioned to drive internal calcium release from the SER spine apparatus. There is feedforward signaling, whereby cAMP–PKA signaling increases internal calcium release, and calcium release drives more cAMP production, thus producing the “genie in the bottle.” cAMP–PKA signaling can also increase calcium flux through voltage-gated calcium channels and NMDAR (not shown). PDE4A provides essential regulation of these feedforward signaling pathways, and is anchored to the SER by DISC1 [68]. Thus, there can be loss of regulation with genetic insults to DISC1, or with inflammation which can similarly unanchor DISC1 (see text). PKA is also anchored to the SER, but by another anchoring protein, AKAP6 (anchoring protein 6). **d** In primate layer III dlPFC, dual immunoEM labeling shows that phosphodiesterase, PDE4A (green arrowheads) and AKAP6 (orange arrowheads), are concentrated in spines on the SER spine apparatus (pink). Adapted with permission from Carlyle et al. [88]. Black arrows indicate the glutamate synapse; ax axon terminal, sp spine, SER smooth endoplasmic reticulum, pseudocolored pink.

cAMP–PKA signaling can also produce transient increases in synaptic strength by increasing transmitter release from the presynaptic terminal, e.g., in chromaffin cells, where PKA phosphorylation primes vesicles for release [63] (Fig. 3a). ImmunoEM and physiological data suggest that this also occurs in primate V1, where the phosphodiesterase, PDE4A, is concentrated in presynaptic terminals, and local stimulation of cAMP signaling increases neuronal firing [30] (Fig. 3b).

### cAMP–PKA signaling in dlPFC

In contrast to V1 where phosphodiesterase 4 (PDE4A) is focused in presynaptic terminals, cAMP-related proteins are concentrated in spines in primate layer III dlPFC, where they are positioned to magnify internal calcium release from the SER (Fig. 3c, d). The SER stores calcium and releases it back into the cytosol via ryanodine receptors (RyR) and IP3 receptors (IP3R), both of which are localized on the SER in the dlPFC [64, 65]. The SER forms a continuous conduit throughout the neuron, and can elaborate extensively when it extends into spines, where it is known as the spine apparatus. As schematically illustrated in Fig. 3c, cAMP–PKA signaling is known to increase calcium release from the SER by phosphorylating RyR and IP3R, while calcium in turn drives cAMP production, thus creating feedforward signaling [66]. This can be particularly destructive when sustained, high levels of cAMP–PKA activity cause PKA phosphorylation of RyR, which causes calcium leak in to the cytosol, e.g., as seen in heart failure [67].

One can see the molecular machinery for cAMP–PKA drive on calcium release from the spine apparatus in layer III dlPFC spines. For example, the anchoring protein, AKAP6, anchors PKA to the SER, and can be seen on the spine apparatus in dlPFC (Fig. 3d). The phosphodiesterase, PDE4A, which catabolizes cAMP, is also localized next to the spine apparatus, and is anchored there by DISC1 [68] (disrupted in schizophrenia 1). Thus, PDE4A is concentrated in axon terminals in V1, but on the spine apparatus in dlPFC, near the glutamatergic synapse.

Transcriptomic analyses of layer III pyramidal cells in rhesus monkey also show an enrichment of genes encoding for calcium channels/transporters and cAMP-activating proteins in dlPFC compared to LIP [24]. dlPFC pyramidal cells have greater expression of the T-type voltage-gated calcium channel, Ca_v3.1_ (*CACNA1G*), the ATPase plasma membrane calcium transporter (*ATP2B4*), and the “master stress” signaling peptide, PACAP (*ADCYAP1*), which drives cAMP and calcium signaling through both Gs and Gq-coupled receptors, (PAC_1_, VPAC_1_, and VPAC_2_). While the location and physiological contributions of these proteins, as well as other calcium channels, are still unknown, the data are consistent with an enrichment of calcium–cAMP-related signaling in dlPFC. Interestingly, a duplication in VPAC_2_ is associated with schizophrenia [69], as discussed below.

**Fig. 4:**
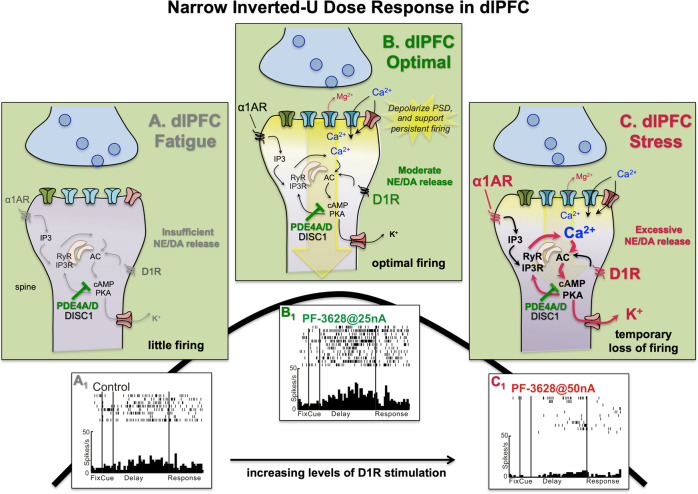
Catecholamines have an inverted-U influence on dlPFC neuronal firing and function. **a** Under conditions of fatigue and/or inadequate catecholamine release in dlPFC, there are low levels of dlPFC neuronal firing and impaired working memory function. Under these conditions, there may be inadequate drive on internal calcium release, as well as inadequate phosphorylation of NMDAR, as described in the text. **b** Optimal levels of catecholamine release in dlPFC enhances delay cell firing in a number of ways. As illustrated here, modest levels of internal calcium release may support persistent firing by depolarizing the PSD, with NE stimulation of α1-AR enhancing IP3R-mediated calcium release, and DA D1R increasing cAMP drive on calcium release. cAMP–PKA signaling also opens nearby K^+^ channels to provide negative feedback to prevent seizures [183] and allow dynamic regulation of network inputs [54]. K^+^ channels include HCN channels which are opened by cAMP and are concentrated on spines in dlPFC [71], but not in V1, where they are expressed in their classic location on distal dendrites [30]. HCN channels in dlPFC behave like K^+^ channels, and may do so by partnering with other K^+^ channels (El Hassar et al. unpublished). The open state of KCNQ2 channels is increased by PKA signaling; these channels are also localized on dlPFC spines [34]. **c** Excessive catecholamine release, as occurs with psychological stress or traumatic brain injury, drives high levels of cAMP–calcium signaling, opening large numbers of HCN and KCNQ channels to reduce neuronal firing. A_1_: An example of a dlPFC delay cell with low levels of persistent firing under control conditions as the monkey performs a working memory task. B_1_: Iontophoresis of a low dose of D1 agonist onto the same neuron enhances its persistent firing during working memory. C_1_: Iontophoresis of a high dose of D1 agonist greatly reduces the persistent firing of the dlPFC delay cell, as likely occurs during uncontrollable stress.

The dendritic spines of layer III dlPFC primate neurons express a large number of receptors that increase (DA D1R, NE α1-AR, M1R, mGluR1, mGluR5) or decrease (NE α2A-AR, mGluR3) feedforward calcium–cAMP signaling, often in close proximity to the spine apparatus [34, 70–74]. These spines also express HCN and KCNQ ion channels whose open state is regulated by cAMP–PKA signaling (Fig. 4). Opening of these K^+^ channels provides negative feedback to prevent seizures, as well as a mechanism to dynamically alter the strength of synaptic inputs onto dlPFC pyramidal cells, which can serve to gate network inputs, and alter dlPFC function based on arousal state [54]. We hypothesize that modest levels of cAMP drive on calcium release near the synapse helps to maintain depolarization of the PSD, but that higher levels open sufficient K^+^ channels to weaken connectivity and reduce firing, thus producing a narrow inverted-U dose–response.

### NE and DA have an inverted-U dose–response in dlPFC via cAMP–calcium signaling

Both NE α1-AR and DA D1R have an inverted-U dose/response on dlPFC persistent firing and working memory function through activation of calcium–cAMP signaling in spines (Fig. 4): moderate levels are essential, but excessive levels reduce firing and cognition through opening of nearby K^+^ channels [53, 72, 74, 75]. Modest levels of stimulation may strengthen persistent firing by magnifying calcium near the PSD (Fig. 4b), and/or by phosphorylation of NMDAR to amplify their synaptic actions [76, 77]. However, high levels of stimulation, as occurs with uncontrollable stress, rapidly reduces neuronal firing and impairs working memory via opening of HCN and KCNQ channels [34, 66, 78, 79] (Fig. 4c). In contrast, high levels of catecholamines strengthen more primitive circuits such as the amygdala [80], switching control of behavior to more unconscious habitual and instinctive responses. This can promote survival, e.g., if one is cut off while driving on the highway, but can be devastating if higher order cognitive evaluation is needed to thrive [81]. With chronic stress exposure, sustained weakening of network connections by calcium–cAMP–PKA–K^+^ signaling leads to removal of spines and dendrites [82–85], a finding also seen in humans [86]. Thus, there are remarkably powerful calcium–cAMP signaling neuromodulatory actions in the dlPFC that can destroy, as well as strengthen, network connections, and which can drive atrophy with chronic stress.

### Regulation of calcium–cAMP signaling in dlPFC

It is essential that these powerful calcium–cAMP–PKA–K^+^ mechanisms are tightly regulated, as they weaken connectivity, but also because their feedforward nature can rapidly generate high levels of cytosolic calcium and cAMP, which can have multiple toxic actions (see below). Regulatory proteins include those that directly bind calcium (i.e., calbindin), as well as those that regulate cAMP signaling. There are high levels of calbindin in layer III pyramidal cells in young monkey [87] and human [28] dlPFC, consistent with the magnification of calcium signaling in these circuits. Proteins that regulate cAMP levels are also concentrated in monkey and human dlPFC. cAMP is catabolized by PDE4. PDE4A and PDE4D are concentrated on the spine apparatus in dlPFC spines [88, 89], e.g., as shown in Fig. 3d above. Proteomic analyses show that PDE4D is one of the few proteins enriched in human dlPFC compared to V1 [46], consistent with the immunoEM in monkey [89].

**Fig. 5:**
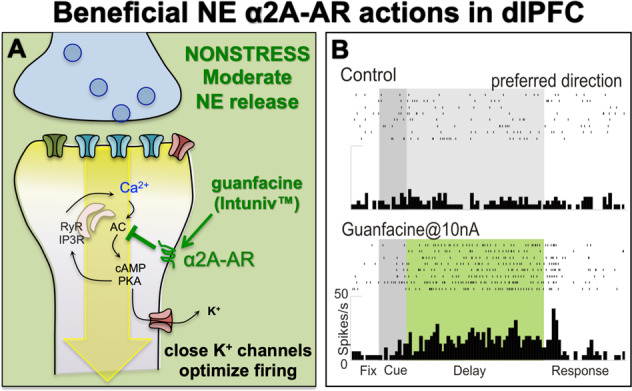
The beneficial effects of NE α2A-AR actions in dlPFC. **a** NE α2A-AR have high affinity for NE, and thus are engaged under conditions of moderate NE release, e.g., nonstress conditions. In layer III dlPFC, α2A-AR are localized postsynaptically on spines, where they inhibit cAMP–calcium signaling and close nearby K^+^ channels to strengthen network connectivity, enhance delay cell firing, and improve a variety of PFC functions in humans, monkeys, and rodents. They also protect PFC circuits from stress exposure (see text). **b** An example of a dlPFC delay cell with weak firing under control conditions, where iontophoresis of guanfacine strongly enhanced task-related firing.

**Fig. 6:**
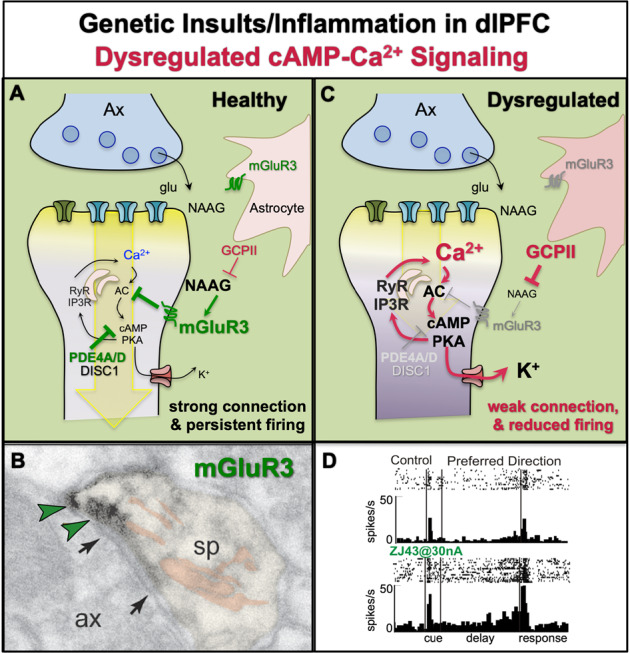
The role of cAMP–calcium dysregulation and inflammation in schizophrenia. **a** Under healthy conditions, feedforward calcium–cAMP signaling in layer III dlPFC spines is held in check by the phosphodiesterases (PDE4s), which catabolize cAMP, and by the G_i/o_-linked receptors mGluR3 (shown) and α2A-AR (not shown), which are both localized on spines and inhibit the synthesis of cAMP. mGluR3 are stimulated by glutamate, but also by NAAG, which is coreleased with glutamate and is selective for mGluR3. mGluR3 are also expressed in their traditional location on astrocytes [73], where they promote glutamate uptake. Astrocytes also synthesize GCPII, an enzyme which catabolizes NAAG and reduces mGluR3 signaling. Under healthy conditions, there are relatively low levels of GCPII, and thus strong NAAG stimulation of mGluR3 on spines, which enhances neuronal firing by inhibiting cAMP–PKA opening of K^+^ channels. **b** ImmunoEM showing mGluR3 labeling (indicated by green arrowheads) on a layer III dlPFC spine in rhesus monkey, next to the glutamate-like synapse (indicated by black arrows) and near the spine apparatus (pseudocolored in pink). Ax axon terminal, sp spine. **c** A variety of environmental and genetic insults associated with schizophrenia effect proteins that play key roles in layer III dlPFC spines. These insults include alterations that weaken NMDAR transmission (which are essential to pyramidal cell firing, but have less influence on interneurons in adult primate dlPFC [184]), as well as insults that weaken the regulation of feedforward calcium–cAMP signaling in spines. When these insults occur early in life (genetic or perinatal insults), they may induce atrophy of higher cortical circuits that are apparent in childhood, adolescence, or young adulthood. These insults may involve inflammatory events, which increase GCPII expression [140], and/or genetic alterations such as insults to *GRM3* (mGluR3) or to *DISC1*, which are associated with increased risk of schizophrenia. Dysregulation of feedforward calcium–cAMP signaling would lead to excessive opening of nearby K^+^ channels, weaker synaptic connectivity and spine loss, and reduction in the persistent firing needed for higher cognition. **d** Iontophoresis of the GCPII inhibitor, ZJ43, onto a dlPFC delay cell enhances the persistent firing during the delay period in a monkey performing a working memory task. Images **b** and **d** were adapted with permission from Jin et al. [73].

There are also receptors that inhibit cAMP production via G_i/o_ signaling: both α2A-AR [71] (Fig. 5a) and mGluR3 [73] (Fig. 6a) are localized on spines in layer III of dlPFC, and both enhance delay cell firing (Figs. 5b and 6d) and working memory performance via inhibition of cAMP–PKA–K^+^ channel signaling. α2A-AR are of interest as they protect PFC spines and working memory from chronic stress [84]. The α2A-AR agonist, guanfacine (Intuniv™) stimulates α2A-AR to enhance dlPFC network connectivity and delay cell firing (Fig. 5), and is now in widespread clinical use based on this research in animals [90].

mGluR3 are a replicated GWAS hit for schizophrenia [91]. mGluR3 are classically located on astrocytes; however, in layer III dlPFC, they are also concentrated on spines (Fig. 6a, b), where they inhibit cAMP–K^+^ channel signaling and enhance delay cell firing [73] (Fig. 6a). mGluR3 are stimulated by both glutamate and NAAG, which is coreleased with glutamate and is selective for mGluR3 [92] (Fig. 6a). NAAG is catabolized by GCPII [93], and iontophoretic inhibition of GCPII enhances delay cell firing in monkeys [73] (Fig. 6d). These beneficial effects appear to be an evolutionary advance, as mGluR3–NAAG–GCPII signaling is associated with higher cognitive function in humans [94], and mGluR3 protein is enriched in human dlPFC vs. V1 [46]. In contrast, mGluR3 are generally presynaptic in rodent cortex [95], and often weaken rather than strengthen synaptic connectivity [96, 97], cautioning that rodent models may be misleading regarding this mechanism.

## Relevance to genetic and inflammatory insults in mental disorders

Genetic and/or environmental insults that dysregulate cAMP–calcium signaling in the dlPFC are often associated with cognitive disorders such as schizophrenia, where dlPFC neuropathology [16, 98], and cognitive deficits including thought disorder [99] are hallmarks of the disease. Loss of dendrites and spines in schizophrenia target deep layer III [16, 98, 100], the same sublayer that contains the microcircuits that generate mental representations [98]. Brain imaging studies of patients in the prodrome to illness show that accelerated gray matter loss in PFC heralds the descent into illness and is associated with signs of inflammation [101]. As reviewed below, inflammatory signaling may share a phenotype with some genetic insults that increase risk of schizophrenia, by reducing the regulation of calcium–cAMP signaling in dlPFC, weakening NMDAR synaptic connections and propelling their removal. Although not at the top of the cortical hierarchy, the PFC may be particularly vulnerable to dysfunction and atrophy given its extensive calcium–cAMP–K^+^ channel signaling, where high levels of calcium–cAMP signaling weaken rather than strengthening network connections.

### Links to NMDAR transmission

It has long been appreciated that reductions in NMDAR signaling mimic or cause psychotic disorders and cognitive deficits (reviewed in [102]). NMDAR antagonists given to healthy subjects can mimic many aspects of schizophrenia, including reduced dlPFC BOLD response during working memory [103], cognitive deficits [104], and psychotic symptoms [105]. A similar profile is seen with autoantibodies against NMDAR [106]. GWAS studies have found linkages between schizophrenia and a variety of glutamate receptors, including *GRIN2A* which encodes for the NMDAR–NR2A subtype [107] Rare *de novo* mutations linked to schizophrenia also include proteins that are part of the NMDAR complex, including loss-of-function mutations in DLG2 [108], a PSD protein that anchors NMDAR–NR2B into the synapse [109].

### Links to calcium channels

GWAS studies have also found links between risk of schizophrenia and alterations in genes encoding for calcium channels [107, 110]. The L-type calcium channel Ca_v1.2_ encoded by *CACNA1C* is a risk factor for multiple mental disorders, and the risk variant of this gene increases channel expression in human brain [111], and increases calcium currents in human-induced neurons [112]. There also linkages with the genes encoding l-type calcium channel subunit *CACNB2*, and the t-type subunit *CACNA1I*. The locations and physiological actions of these channels are not yet known in nonhuman primate dlPFC, but the risk allele in *CACNA1C* is associated with inefficient dlPFC activation in humans [111]. It is also of interest that *de novo* mutations in the internal calcium channel, RyR types 3 or 2, are associated with increased risk of adult-onset [108] or child-onset [113] schizophrenia, respectively, suggesting that mutations that bring excessive amounts of calcium into the neuron (*CACNA1C*), or those that alter internal calcium release (*RYR2, RYR3*), confer risk for mental illness.

### Links to cAMP signaling

Genetic mutations that increase cAMP signaling are also associated with schizophrenia. A rare copy number variant that duplicates the gene encoding for VPAC_2_ increases cAMP signaling and increases risk of schizophrenia [69, 114]. Thus, it will be important to learn how VPAC_2_ stimulation influences dlPFC physiology, and whether excessive VPAC_2_ stimulation reduces dlPFC neuronal firing by opening K^+^ channels in spines.

There are also important genetic links with schizophrenia risk and proteins already known to inhibit cAMP signaling in dlPFC spines, including mGluR3 (*GRM3*) and *DISC1*, which normally serves to anchor PDE4s to the spine apparatus.

#### mGluR3 signaling

*GRM3* is a replicated GWAS hit in schizophrenia [91], but its relationship to cognitive dysfunction had been a mystery, as mGluR3 were generally considered to be primarily expressed in astrocytes. It is now appreciated that mGluR3 are concentrated on spines as well as astrocytes in layer III dlPFC, where they strengthen synaptic connectivity and enhance delay cell firing by inhibiting cAMP–K^+^ channel signaling [73] (Fig. 6a). An example of mGluR3 labeling next to the synapse and the SER spine apparatus on a dendritic spine in layer III dlPFC is shown in Fig. 6b [73]. Thus, insults that diminish mGluR3 signaling, e.g., by decreasing mGluR3 expression or function, or by increasing the expression of GCPII and thus reducing NAAG actions at mGluR3 [115], would weaken dlPFC connectivity, as schematized in Fig. 6c. This hypothesis is supported by physiological data from monkeys, where local inhibition of GCPII enhances delay cell firing [73] (Fig. 6d). Analyses of the dlPFC from patients with schizophrenia show reduced mGluR3 and increased GCPII expression [116], and genetic alterations in *GRM3* are associated with impaired dlPFC cognitive function [117, 118]. Most recently, genetic variation in this pathway has been related to human intelligence in healthy subjects, where an alteration in the gene encoding for GCPII that increases its expression causes a corresponding decrease in NAAG levels in brain associated with inefficient dlPFC activation and reduced IQ [94]. This is a rare example where intelligence within the normal range can be linked to a single gene, emphasizing the power of NAAG-mGluR3 signaling in the human association cortex.

#### DISC1

A rare loss-of-function translocation in the gene encoding for DISC1 is associated with high rates of mental illness in a large British family [119]. This genetic insult is associated with a variety of mental disorders –schizophrenia, bipolar disorder, depression, and ADHD—all of which involve PFC dysfunction [120]. DISC1 is an anchoring protein [121, 122], and is important for brain development [123] and regulating the NMDAR PSD to maintain spine integrity [124]. In monkey dlPFC, DISC1 anchors PDE4A to the spine apparatus [68] (Fig. 6a), where it is positioned to regulate cAMP drive on internal calcium release. Thus, loss of DISC1 should dysregulate this signaling pathway, and should be particularly deleterious under conditions of stress when there are high levels of cAMP–calcium signaling and a greater need for PDE4 catabolism (schematized in Fig. 6c). This hypothesis is supported by data from rats with viral knockdown of DISC1 in the mPFC, which had little effect under basal conditions, but lowered the threshold for stress-induced working memory deficits [125]. Similar effects may occur in humans, where those with more protected lives would have less impact of DISC1 translocation, while those with greater stress exposure may have substantial PFC deficits resulting in a psychiatric diagnosis. This is an example of how genetic alterations may interact with the environment to confer risk for PFC dysfunction and mental illness. There are also interactions between genes, e.g., where multiple insults in genes with related functions may add together to increase risk. For example, genetic variations in the genes encoding for DISC1, mGluR3, COMT, and RGS4 interact to increase risk of schizophrenia [126], and these proteins are all found in primate layer III microcircuits in dlPFC [68, 73, 127, 128].

### Dysregulated calcium signaling induces inflammation

The genetic changes described above would elevate cytosolic calcium, which can have a number of detrimental actions. One known result of elevated cytosolic calcium is the induction of an inflammatory response via calcium overload of mitochondria [129–131]. Although most in vitro studies of calcium toxicity have used very high levels of excitotoxins that induce apoptosis, e.g., as occurs with stroke, most cognitive disorders do not show evidence of apoptotic cell death, but rather, atrophy of dendrites and spines (e.g., schizophrenia and AD) [16, 132], and/or autophagic neurodegeneration (e.g., in AD) [133]. These changes likely arise from sustained, elevated levels of cytosolic calcium that are not high enough to induce apoptosis, but sufficient to induce inflammatory responses such as activation of complement (C1q, C3, C4a) expression, which tags synapses for removal by microglia [134]. Importantly, genetic insults that increase the activity of C4a have the greatest incidence of increasing risk for schizophrenia, emphasizing the importance of this final common pathway to cognitive disorder [135]. Thus, atrophy may occur through both direct genetic insults that increase complement signaling (e.g., C4a), or through indirect genetic insults that result in dysregulated calcium signaling that induce inflammatory responses and lead to a similar phenotype.

### Inflammation dysregulates cAMP–calcium signaling and can mimic genetic insults

While calcium dysregulation drives inflammatory cascades, the converse is also true, where inflammation can dysregulate cAMP–calcium signaling, thus creating a vicious cycle that can propel atrophy. Inflammatory cascades can induce a number of changes that can weaken dlPFC function and mimic the effects of genetic insults. For example, MK2 inflammatory signaling inactivates and unanchors PDE4, so that it may no longer be correctly positioned to inhibit cAMP drive on calcium release [136, 137], potentially mimicking *DISC1* translocation. Inflammation also increases the expression of agents that reduce dlPFC network connectivity and neuronal firing, e.g., increasing (1) GCPII [138–140] (Fig. 6c), which may mimic genetic insults to *GRM3*, and (2) kynurenic acid (KYNA) [141], which blocks NMDAR [142, 143] and Nic-α7R [144], and may mimic multiple genetic insults that reduce NMDAR signaling. It is noteworthy that patients with schizophrenia have elevated KYNA levels in dlPFC and plasma, related to signs of elevated inflammation and impaired dlPFC structure and function [143, 145], supporting this view.

An extensive literature indicates that environmental insults *in utero* may sensitize inflammatory signaling and increase risk of later mental illness, e.g., by exacerbating the pruning of dendritic spines later in adolescence [146]. Animal models show that maternal infection increases the expression of GCPII [140] and complement C1q [147] in the brains of offspring, and conversely, mothers with high plasma C1q during pregnancy have an increased risk of having a child who later has schizophrenia [148]. Thus, genetic insults, e.g., to C4a [135], or environmental insults that sensitize inflammatory signaling [146], may lead to a similar phenotype of aggravated spine loss. Interestingly, severe childhood abuse is associated with schizotypal cognitive deficits and thought disorder [149], suggesting that activation of stress signaling pathways by psychological events may also play a role in sensitizing the system to dysfunction.

## Relevance to age-related cognitive disorders

While critical genetic and perinatal insults often express as disease relatively early in life, more subtle genetic changes and/or environmental insults in adulthood may only manifest as their consequences build over a long lifetime. Activation of inflammatory and stress signaling pathways play a major role in age-related cognitive disorders, e.g., where psychological stress [150] and inflammatory insults such as head injury and diabetes [151] increase the risk of the sporadic, very common form of AD. There is now extensive evidence that dysregulated calcium signaling is an early etiological factor in AD pathology [152–156], and can be seen in aging entorhinal neurons that are most vulnerable to AD pathology [65, 157]. Regulation of calcium and cAMP signaling is reduced with advancing age [28, 88, 158], leading to increased calcium leak into the cytosol from PKA-phosphorylated pRyR [65, 159], and reduced calcium binding in the cytosol as the calcium binding protein, calbindin, is lost with age [28, 88, 158]. Elevated cytosolic calcium contributes to a range of detrimental actions in association cortex and hippocampus, and increased risk of AD.

**Fig. 7:**
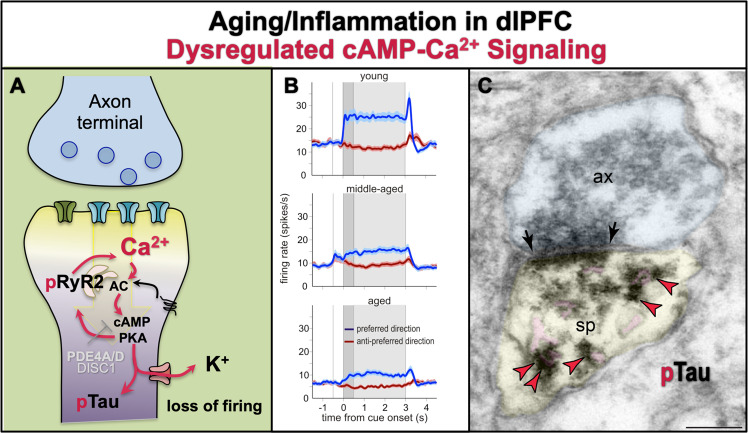
The role of cAMP–calcium dysregulation in age-related pathology. **a** With advancing age, there is loss of PDE4 expression from dlPFC spines [88], and increased calcium–cAMP signaling which reduces neuronal firing via excessive opening of K^+^ channels [162]. The increase in cytosolic calcium would be particularly detrimental when the calcium binding protein, calbindin, is lost from pyramidal cells, but not interneurons, with increased age. Gradual increases in cytosolic calcium over a long lifetime may have a number of toxic actions, including increasing tau phosphorylation (pTau) [65, 88], amyloid deposition, neuroinflammation, and ultimately, neurodegeneration. **b** The persistent neuronal firing of dlPFC delay cells from young adult, middle-aged, and aged monkeys performing a working memory task. The spatial cue is presented at 0 s for 0.5 s (dark gray), while the delay period ensues for the following 2.5 s (light gray shading), followed by the saccadic response to the remembered location. Strong working memory requires high levels of persistent firing for the neuron’s preferred direction (blue) compared to its nonpreferred directions (red) across the entire delay period. There was a highly significant reduction in persistent firing with advancing age, beginning in middle age when PFC cognitive deficits become evident in both monkeys and humans. Firing was restored by local inhibition of feedforward calcium–cAMP–K^+^ signaling. **b** was adapted with permission from Wang et al. [162]. **c** With advancing age, there is a large increase in PKA phosphorylation of tau at serine-S214, which collects on microtubules and the SER [65, 88]. This image shows pS214Tau (indicated by red arrowheads) aggregating on the SER spine apparatus (pink pseudocolor) within a dendritic spine from layer III dlPFC in an aged monkey. Ax axon terminal, sp spine, synapse indicated by black arrows; scale bar = 200 nm. Image from D. Datta.

Age-related deficits in dlPFC and medial temporal cortical cognitive functions can be seen in rodents, monkeys and humans (reviewed in [160]), with deficits in dlPFC executive functions beginning in middle age [161]. Delay cell persistent firing shows a similar loss of firing in middle and advanced age, due to excessive cAMP–calcium opening of K^+^ channels [162] (Fig. 7a, b). Parallel findings are seen in aged rodent hippocampus, where dysregulated calcium signaling opens SK K^+^ channels and reduces neuronal firing [163]. Excessive cAMP–calcium signaling also drives tau phosphorylation in the aging monkey dlPFC, where PKA-phosphorylated tau aggregates on the SER (Fig. 7a, c) and on microtubules, priming tau for hyperphosphorylation by GSK3β (discussed below).

Excessive cytosolic calcium also induces calcium overload of mitochondria that can initiate inflammatory signaling, and under extreme conditions, apoptosis [129–131]. The data suggest that sustained, moderate levels of calcium dysregulation contribute to mitochondrial abnormalities [164, 165] and inflammation [166] with advancing age in the primate dlPFC. Complement C1q is expressed in aged monkey dlPFC near dysmorphic mitochondria and near synapses on spines [166], which is a likely forerunner to age-related loss of spines [167]. These age-related changes are amplified in AD [132, 168, 169], where loss of synapses correlates with cognitive deficits [132].

### Increased AD pathology

There is a longstanding hypothesis that dysregulated calcium signaling drives neuropathology in AD [152–156]. For example, high levels of cytosolic calcium increase the activity of BACE, the critical enzyme for cleavage of APP to Aβ [170]. As described above, calcium dysregulation also drives inflammatory processes that lead to synapse loss. Importantly, high levels of cytosolic calcium can activate calpain, which is highly expressed in AD cortex in correspondence with pathology [171–173], and can drive both tau hyperphosphorylation and neurodegeneration. Calpain cleaves and disinhibits GSK3β to hyperphosphorylate tau to form neurofibrillary tangles [174, 175]. Calpain also cleaves heat shock protein Hsp70.1 to drive autophagic neurodegeneration [133]. Thus, excessive calcium signaling can increase all the key features of AD pathology.

There is a striking correspondence between tau pathology and calcium usage across the cortical hierarchy. Tau pathology and neurodegeneration in AD specifically afflicts glutamatergic neurons in the association cortex, starting in the perirhinal and entorhinal cortices (layer II), then expanding more generally in pyramidal cells of the association cortices, with primary sensory cortices only afflicted at end-stage disease [12, 13, 15, 176]. The expression of the calcium binding protein, calbindin, in pyramidal cells shows a similar relationship, with the most expression in cortical areas at the top of the hierarchy, and the least in primary sensory cortices [47, 177], consistent with the hypothesis that neurons engaged in higher cognitive operations utilize high levels of calcium. However, loss of calbindin with advancing age renders these cells particularly vulnerable to calcium’s toxic effects on tau phosphorylation and neurogeneration, as these are the cells afflicted in AD [28, 178]. Overall, these data suggest that neurons that require high levels of internal calcium signaling for their normative functioning must express correspondingly high levels of calbindin in their cytosol for adequate protection, and that loss of calbindin with stress and/or age confers tremendous vulnerability to amyloid and tau pathology, atrophy, and/or neurodegeneration depending on the subcellular location(s) of the calcium dysregulation, and the time course of the dysregulating events [179].

## Summary

In summary, evidence from multiple sources indicates that elevated calcium signaling occurs in higher cortical circuits where it may be needed to maintain depolarization of the PSD to support the persistent firing that underlies much of higher cognitive functioning, and to dynamically gate network connections. In particular, the magnification of internal calcium release by cAMP signaling requires tight regulation, given its feedforward signaling. Thus, the “genie” that sparks our remarkable cognitive abilities also renders us vulnerable to cognitive disorders when regulatory proteins are unanchored or lost, and the “genie” can no longer be put back in the “bottle.” Dysregulated calcium–cAMP signaling can lead to a number of detrimental actions, including excessive opening of K^+^ channels that reduce neuronal firing, mitochondrial changes that drive inflammation and loss of synapses, and activation of kinases that induce tau pathology and degeneration. Therapeutic strategies that help to restore calcium regulation may protect higher cortical circuits and reduce the risk of cognitive disorders.
